# Supplementary material for: The interactome of intact mitochondria by cross-linking mass spectrometry provides evidence for coexisting respiratory supercomplexes
Source: Mol Cell Proteomics. 2017 Dec 8;17(2):216–32. doi: 10.1074/mcp.RA117.000470 (PMC5795388; doi:10.1074/mcp.RA117.000470)
Supplement: Supplemental Data [file supp_RA117.000470_133922_0_supp_23978_fzffwf.zip › spectra_annotation/mito_spectra_annotation/239-2-3-1-9-1.pdf]

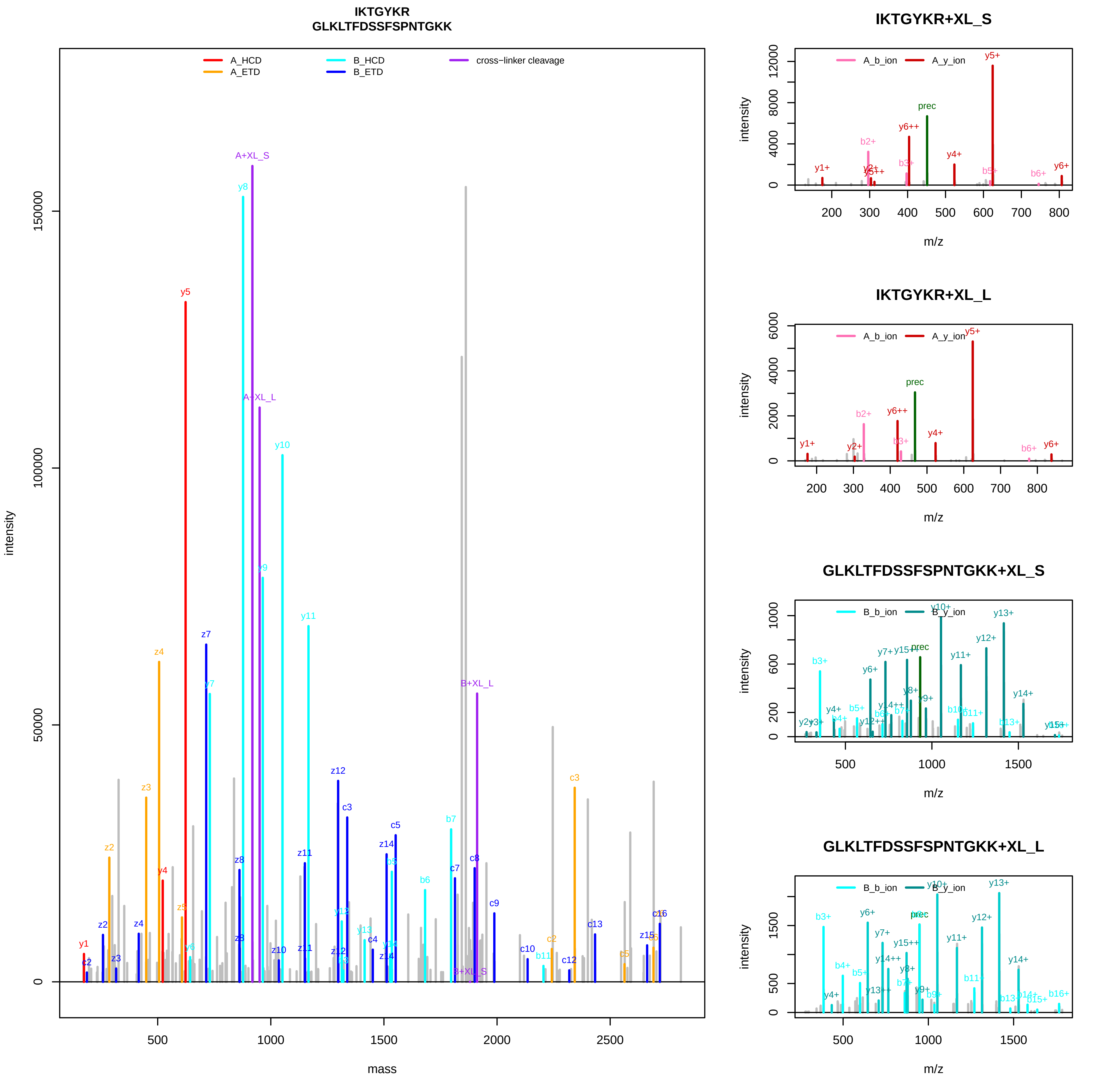
IKTGYKR
GLKLTFDSSFSPNTGKK
A_HCD
A_ETD
B_HCD
B_ETD
cross−linker cleavage
intensity
mass
0
50000
100000
150000
500
1000
1500
2000
2500
A+XL_S
A+XL_L
B+XL_L
y1
y4
y5
y6
y7
y8
y9
y10
y11
y13
y14
z2
z3
z4
z5
z7
z8
z10
z11
z12
z14
z15
c2
c3
c4
c5
c7
c8
c9
c10
c12
c13
c16
b6
b7
b11
IKTGYKR+XL_S
A_b_ion
A_y_ion
prec
y1+
y2+
y4+
y5+
y6+
y6++
b2+
b3+
b5+
b6+
m/z
IKTGYKR+XL_L
GLKLTFDSSFSPNTGKK+XL_S
B_b_ion
B_y_ion
b3+
b4+
b5+
b7+
b10+
b11+
b13+
y6+
y7+
y8+
y9+
y10+
y11+
y12+
y13+
y14+
y15++
GLKLTFDSSFSPNTGKK+XL_L
b16+
